# Polidocanol-foam treatment of varicose veins: Quality-of-life impact compared to conventional surgery

**DOI:** 10.1016/j.clinsp.2024.100346

**Published:** 2024-04-04

**Authors:** Lissa Severo Sakugawa, Felipe Soares Oliveira Portela, Andressa Cristina Sposato Louzada, Maria Fernanda Cassino Portugal, Marcelo Passos Teivelis, Cynthia de Almeida Mendes, Lucas Lembrança Pinheiro, Marcelo Fiorelli Alexandrino da Silva, Alexandre Fioranelli, Nelson Wolosker

**Affiliations:** a Hospital Israelita Albert Einstein, São Paulo, SP, Brazil; b Faculdade Israelita de Ciências da Saúde Albert Einstein, São Paulo, SP, Brazil; c Santa Casa de Misericórdia de São Paulo, São Paulo, SP, Brazil

**Keywords:** Varicose veins, Saphenous vein, Polidocanol, Sclerotherapy, Quality of life

## Abstract

**Background and objective:**

Lower limb varicose veins are a prevalent disease associated with several available treatment options, including conventional surgery and polidocanol foam sclerotherapy. However, few studies have analyzed therapeutic modality outcomes based on Patient-Reported Outcome Measures (PROMs). This large sample-size study was designed to evaluate the outcomes of polidocanol foam sclerotherapy compared to conventional surgery based on an analysis of PROMs.

**Methods:**

This was a prospective, observational, and qualitative study of 205 patients who underwent varicose vein treatment with either polidocanol foam sclerotherapy (57 patients, 90 legs) or conventional surgery (148 patients, 236 legs). Patients were preoperatively assessed and re-evaluated 30 days after the procedure using the Venous Disease Severity Score (VCSS) and specific venous disease quality-of-life questionnaires (VEINES-QoL/Sym).

**Results:**

Both treatments significantly improved VCSS and VEINES results 30 days after the procedure (p < 0.05). However, surgery promoted greater improvements in VCSS (on average 4.02-points improvement, p < 0.001), VEINES-QoL (average 8-points improvement, p < 0.001), and VEINES-Sym (average 11.66 points improvement, p < 0.001) than did sclerotherapy. Postoperative pain and aesthetic concerns about the legs were the domains of the questionnaires in which the results varied the most between the treatment modalities, with worse results for sclerotherapy.

**Conclusion:**

Both polidocanol foam sclerotherapy and conventional surgery positively impact patients’ quality of life after 30 days, but the improvement is more significant for patients who undergo conventional surgery.

## Highlights

• Both polidocanol foam sclerotherapy and conventional surgery had positive impacts on patient quality of life.
• Conventional surgery promotes greater improvement in patient quality-of-life than sclerotherapy.
• Post-procedure pain and aesthetic concerns about the legs worsen the efficacy of sclerotherapy.

## Introduction

Lower limb Varicose Veins (VVs) are permanently dilated and tortuous veins that affect up to 50 % of men and 70 % of women. They can cause mild symptoms such as fatigue, a feeling of heaviness or burning, swelling, and itching in the legs or more severe complications such as phlebitis, ulcers, and bleeding.^1^

Due to the high incidence of VVs, the number of surgeries for the treatment of VVs is substantial, reaching 80,000 per year in England and 70,000 per year in Brazil.^2^^,^^3^ The conventional surgical approach is the most widely used treatment in clinical practice and yields excellent results, which is why it is the preferred method in the Brazilian public health system. Alternatives such as the injection of sclerosing substances and the use of LASER or radiofrequency ablative techniques are also available.^4^

Assessing the improvement in VV symptoms is complex, as VV symptoms are not pathognomonic, have poor correlations with anatomical and imaging parameters, and are strongly associated with psychological aspects.^5^ A robust correlation between quality of life and the clinical outcome of surgical VV treatment has already been demonstrated.^6^

In recent decades, healthcare providers have increasingly recognized the importance of using patient-centered tools for various health conditions, including varicose veins, to offer better treatments in terms of quality, efficacy, and cost-effectiveness^7^ Patient-reported outcome Measures (PROMs) are instruments that collect information on symptoms, functional status, and quality of life, making it possible to define the success of therapeutic interventions based on patient experience.^8^

However, in the scientific literature, the definition of success in treating VVs is still fundamentally based on technical criteria.^9^^,^^10^ Few studies have analyzed PROMs to determine the effectiveness of treatment, and several of them use generic questionnaires that are not specific to venous disease.^11^^,^^12^ Additionally, few studies of polidocanol have addressed quality of life,^13^^,^^14^ and most studies include few patients, if any, with more advanced degrees of Chronic Venous Disease (CVD).^14^^,^^15^ No impact studies use PROMs in low- or middle-income countries. In this large sample (205 patients) of patients involving PROMs, the authors aimed to evaluate the impact of treating CVD with Polidocanol Foam Sclerotherapy (PFS) compared to Conventional Surgery (CS).

## Methods

### Study design

This prospective, observational, and qualitative study involved 205 patients who underwent VV treatment. Among them, 57 patients underwent polidocanol sclerotherapy (foam group), and 148 patients underwent conventional surgical treatment (surgical group); these patients were the control group. The study took place at two institutions within the public health system in the city of São Paulo, Brazil, from October 2021 to October 2022. Patients were consecutively enrolled.

This research was approved by the ethics committees of the respective institutions according to the following protocols: CAAE 51668821.6.0000.0071 and 51668821.6.3001.0083. A consent form was administered to all patients who agreed to participate in the study. Patients who underwent PFS received treatment on an outpatient basis. The tributary veins were punctured under direct vision, and 1 % polidocanol foam was applied. In cases where the saphenous veins were insufficient, ultrasound-guided distal puncture was performed, followed by the injection of 3 % polidocanol foam.^16^ Patients who underwent CS were treated in an operating room under spinal anesthesia. The VV was resected through staggered incisions. In patients with great saphenous vein insufficiency, treatment was performed through an incision at the root of the thigh, with ligation of tributary veins and resection of the insufficient segment with a pin-stripper.

Two occasions were assessed for each patient: before the procedure of choice and again 30 days after the treatment. During both assessments, the Clinical manifestations, Etiologic factors, Anatomic distribution of disease, and Pathophysiologic findings (CEAP) were evaluated, and the Venous Clinical Severity Score (VCSS)^17^ and VEINES/QOL-Sym^18^ (Portuguese version)^19^ questionnaires were applied. In terms of the CEAP classification, the primary focus was on the assessment of clinical manifestations (C), by which the status of venous disease is stratified into seven categories:

• C0: No signs of venous disease;
• C1: Telangiectasias or reticular veins;
• C2: Visible varicose veins;
• C3: Edema;
• C4: Skin changes ‒ hyperpigmentation, eczema, lipodermatosclerosis;
• C5: Healed ulcer.
• C6: active ulcer

The VCSS (Appendix 1) comprises 10 attributes (pain, varicose veins, edema, pigmentation, inflammation, induration, number of ulcers, duration of ulcers, size of ulcers, and compressive therapy), each rated on a scale of 0 to 3, representing absent, mild, moderate or severe symptoms, respectively.^20^ In this scenario, the higher the score, the more severe the venous disease. All these attributes were analyzed in this study.

The VEINES/QOL-Sym (Appendix 1) is a specific quality-of-life assessment tool for venous disease that consists of 26 items. These items include questions about symptoms (10 items), limitations in daily activities (9 questions), psychosocial impact (5 items), changes in complaints over 1 year (1 item), and the time of day when symptoms are most frequent.^21^ In these cases, the higher the score, the better the patient's quality of life. To facilitate a more comprehensive analysis of changes in quality of life before and after treatment, the VEINES scores were transformed into a 0‒100 scale using a method previously described in the literature.^22^ All items from the original questionnaire were evaluated both before and 30 days after each type of procedure. The demographic data of patients who underwent either CS or PFS and their preoperative CEAP classification were examined. The VCSS and VEINES/QOL-Sym scores were computed before and after the treatment, and comparative analyses were conducted between these periods and the two treatment groups.

Furthermore, the authors investigated the factors within each score that had the most significant impact on the differences between the treatments. To achieve this, the authors calculated the average change in each score associated with each type of procedure, and linear regression analysis was employed to identify the items from each questionnaire that exhibited statistically significant effects.

### Sample calculation and statistical analysis

Based on the main objective, which was to find a difference of at least 1.5 points in the VCSS between the CS and PFS groups since, in a reference study, the average variability in the VCSS was 2.355 points,^23^ with 80 % power and 95 % Confidence, the sample size required for the study included at least 39 patients in each group, considering a two-tailed test.

All the statistical analyses were performed using SPSS software version 22 (IBM ‒ Armonk, New York, USA). The patients' clinical and epidemiological characteristics were analyzed, as were all the variables in each of the quality-of-life scores (VCCS, VEINES-QoL, and VEINES-Sym).

Student's *t*-test was used to assess continuous variables, while the Chi-Square test was used to analyze categorical variables.

To analyze the variations in the quality-of-life scores at each time point (pre- and posttreatment), a generalized estimating equation with a normal distribution and identity link function was used, assuming an AR(1) correlation matrix between the time points and Bonferroni multiple comparisons. Multiple linear regression was used in the multivariate analysis.

A p-value less than or equal to 0.05 was considered indicative of statistical significance for all tests.

## Results

In total, 57 patients (involving 90 limbs) were treated with PFS, while 148 (involving 236 limbs) underwent CS.

The demographic data for both groups are presented in Table 1. Patients who underwent PFS were significantly more likely to have hypertension (58.8 % vs. 29.1 %, p < 0.001), diabetes (29.4 % vs. 8.1 %, p < 0.001), or obesity (48 % vs. 24.2 %, p < 0.001). Both groups were predominantly composed of females, with no significant difference between them (p = 0.121). Patients in the foam group were significantly older (p = 0.002) and had a greater body mass index (p < 0.001).

**Table 1:** Demographics and clinical characteristics of the sample.

		Group				Total		p-value
		CS		PFS				
		n	%	n	%	n	%	
**Average age (years)**		56.7		62.8				0.002^d^
**Gender**	Female	112	75.7	37	64.9	149	72.7	0.121ª
	Male	36	24.3	20	35.1	56	27.3	
**Hypertension**		43	29.1	30	58.8	73	36.7	**<0.001ª**
**Diabetes**		12	8.1	15	29.4	27	13.6	**<0.001ª**
**Smoking**		13	8.8	3	6.3	16	8.2	0.765^b^
**Physical activity**		38	25.9	14	31.1	52	27.1	0.487^a^
**BMI** Average (kg/m^2^)		27.0		31.3				**<0.001^c^**
Underweight/Eutrophic		51	35.2	13	26	64	32.8	
Overweight		59	40.7	13	26	72	36.9	
Obesity class 1		31	21.4	11	22	42	21.5	
Obesity class 2		3	2.1	9	18	12	6.2	
Obesity class 3		1	0.7	4	8	5	2.6	

Statistical tests: Chi-Square (a); Fisher's test (b); likelihood ratio (c); Student's *t*-test (d).

CS, Conventional Surgery; PFS, Polidocanol Foam Sclerotherapy; BMI, Body Mass Index.

The CEAP classification of the treated limbs is detailed in Table 2. While the foam group did have a greater percentage of patients with more advanced stages of CVD (24.4 % of individuals with CEAPs C5 and C6, compared to 9.3 % of patients in the control group), there was no statistically significant difference between the groups (p = 0.455).

**Table 2:** Preoperative CEAP classification of the treated limbs.

	PFS	CS	p value
**C1**	7 (7.7 %)	0	0.455^a^
**C2**	18 (20.0 %)	38 (16.1 %)	
**C3**	23 (25.5 %)	60 (25.4 %)	
**C4**	20 (22.2 %)	116 (49.1 %)	
**C5**	9 (10.0 %)	0	
**C6**	13 (14.4 %)	22 (9.3 %)	
**Total**	90	236	

Statistical test: Chi-square test for trends (a).

CS, Conventional Surgery; PFS, Polidocanol Foam Sclerotherapy.

A comparison of the VCSS and VEINES scores before and after treatment for both groups is shown in Tables 3 to 5.

**Table 3:** Scores (mean ± standard deviation) and comparisons between groups (CS and PFS) at the pretreatment time point.

	Pretreatment values					
	CS	PFS	Mean difference (CS–PFS)	p	95 % CI	
					Inferior	Superior
**VCSS**	18.04 ± 4.52	20.33 ± 6.53	-2.29	0.019	-4.35	-0.24
**VEINES-QoL**	64.62 ± 11.12	64.48 ± 11.02	0.24	>0.999	-4.22	4.69
**VEINES-Sym**	61.93 ± 15.78	59.29 ± 15.57	2.81	>0.999	-3.21	8.83

CS, Conventional Surgery; PFS, Polidocanol Foam Sclerotherapy; 95 % CI, Confidence Interval.

For the statistical analysis, a generalized estimating equation with a normal distribution and identity link function was used, assuming an AR(1) correlation matrix between the time points and Bonferroni multiple comparisons.

Before treatment (Table 3), patients who were treated with PFS had notably greater mean VCSS than those who underwent CS (20.33 vs. 18.04, p = 0.019). However, there was no difference between the two groups in the pre-procedure assessment concerning the VEINES scores for quality of life (control 64.62 vs. foam 64.48, p > 0.999) or for symptoms (control 61.93 vs. foam 59.29, p > 0.999).

Both treatments (Table 4) resulted in a significant improvement in scores 30 days after the procedures (p ≤ 0.05 in all patients).

**Table 4:** Comparison of pre- and posttreatments.

	CS mean difference (pre–post)	p	95 % IC		PFS mean difference (pre–post)	p	95 % IC	
			Inferior	Superior			Inferior	Superior
**VCSS**	+3.86	<0.001	2.76	4.97	+2.13	0.005	0.44	3.82
**VEINES-QoL**	-15.58	<0.001	-18.98	-12.19	-7.81	<0.001	-12.84	-2.78
**VEINES-Sym**	-20.45	<0.001	-25.04	-15.85	-11.60	<0.001	-18.40	-4.79

CS, Conventional Surgery; PFS, Polidocanol Foam Sclerotherapy; 95 % CI, Confidence Interval.

For the statistical analysis, a generalized estimating equation with a normal distribution and identity link function was used, assuming an AR(1) correlation matrix between the time points and Bonferroni multiple comparisons.

Nevertheless, the surgical group exhibited a greater improvement rate than the foam group did (Table 5). Specifically, the post-treatment VCSS of patients who underwent CS was, on average, 4.02 points lower (indicating better results) than that of patients who underwent PFS (14.48 vs. 18.50, p < 0.001). Concerning the VEINES scores, patients in the surgical group scored an average of 8 points higher in terms of quality-of-life (80.09 vs. 71.99, p < 0.001) and an average of 11.66 points higher in terms of symptoms (82.11 vs. 70.50, p < 0.001) than did those in the foam group.

**Table 5:** Post-treatment scores (mean ± standard deviation) and comparisons between groups (CS and PFS).

	Posttreatment values					
	CS	PFS	Mean difference (CS–PFS)	p	95 % CI	
					Inferior	Superior
**VCSS**	14.48 ± 2.66	18.50 ± 7,09	-4.02	<0.001	-6.35	-1.70
**VEINES-QoL**	80.09 ± 9.29	71.99 ± 11,28	8.01	<0.001	2.81	13.21
**VEINES-Sym**	82.11 ± 10.56	70.50 ± 14,80	11.66	<0.001	4.64	18.69

For the statistical analysis, a generalized estimating equation with a normal distribution and identity link function was used, assuming an AR(1) correlation matrix between the time points and Bonferroni multiple comparisons

CS, Conventional Surgery; PFS, Polidocanol Foam Sclerotherapy; 95 % CI, Confidence Interval.

Subsequently, the authors analyzed all criteria within each questionnaire to determine which factors most significantly contributed to the superior performance of surgery compared to foam treatment. Pain emerged as the primary factor that substantially contributed to the significantly improved postoperative VCSS in the control group compared to the foam group. Although the groups initially exhibited similarities concerning this symptom (p > 0.999, 95 %CI -0.56; +0.83), patients who underwent CS experienced a significant improvement in this regard 30 days after the procedure (mean improvement in VCSS of 1.28 points, p < 0.001, 95 % CI +0.80; +1.77), whereas patients who underwent PFS did not exhibit a statistically significant improvement (mean improvement in VCSS of 0.5 points, p = 0.642, 95 % CI -0.32; +1.34).

According to the VEINES-Sym questionnaire, the scores in the domains related to edema and the sensation of heat or burning did not significantly improve in the foam group compared to the control group, leading to less favorable postoperative scores. Concerning edema, there was a mean difference of 0.95 points (p < 0.001, 95 % CI -1.33; -0.57) in the postoperative score favoring the surgical group. Regarding the sensation of burning, there was an average improvement of 1.31 points in the surgical group (p<0.001, 95 % CI -1.99; -0.63), whereas the foam group exhibited only a minor and nonsignificant improvement of 0.24 points (p > 0.999, 95 % CI -1.19; +0.71).

In the context of the VEINES-QoL questionnaire, concerns about the risk of falling and the impact of leg appearance on clothing choice were the factors that led to a less favorable postprocedure evaluation in the foam group than in the control group. Treatment with CS resulted in an average improvement of 1.78 points in patients’ concerns about the risk of falling (p < 0.001, 95 % CI -2.49; -1.06), while there was a slight and nonsignificant worsening of 0.16 points (p > 0.999, 95 % CI -0.71; +1.03) after PFS. Regarding the influence of leg appearance on clothing choice, surgery led to a significant average improvement of 2.05 points (p < 0.001, 95 % CI -2.73; -1.38), whereas foam treatment brought about a slight nonsignificant improvement of 0.45 points (p > 0.999, 95 % CI -1.40; +0.49).

According to multivariate analysis (see Appendix 2), regarding the VCSS, a higher preoperative (baseline) score was associated with a less favorable post-treatment outcome in the foam group than in the control group (p < 0.001). Regarding the VEINES scores, grade 2 obesity significantly influenced the foam group's less favorable results 30 days after the procedure, both in terms of symptoms and quality of life (p < 0.001 in both scenarios).

## Discussion

While interventions for treating CVD are commonly performed in clinical practice, the outcomes of these interventions have been inconsistent.^24^ Most studies primarily evaluate the success of VV treatment based on technical criteria, such as the presence of venous reflux and remaining veins, with limited consideration of clinical criteria related to patients’ symptoms and quality of life.^25^

Quality-of-life questionnaires and PROMs serve as valuable tools for evaluating the impact of VVs and their treatment on patients' well-being.^26^ It is important to note that no perfect scoring system is available to comprehensively assess the clinical complexity and patient perceptions of venous disease. In this study, for a more thorough analysis, the authors chose to combine a scoring system focused on clinical evaluation (VCSS) with one focusing on the patient's perceptions of their Symptoms (VEINES-Sym) as well as the impact of these symptoms on their Quality-of-Life (VEINES-QoL). The VCSS enables the measurement of subtle changes in the severity of CVD. Nevertheless, it encompasses many symptoms not specific to VVs and may not be particularly useful, especially for patients with lower CEAP classifications (C1 to C3). On the other hand, the VEINES questionnaire is currently one of the most reasonable options for assessing the impact of CVD on quality of life across the entire spectrum of CVD.^27^ However, the ability of the original VEINES scoring system to evaluate changes in quality of life between two different time points may be limited. For this reason, the authors chose to use an alternative approach, as previously described in the literature, to adapt this scoring system objectively.^21^

In this study, 44 legs (13.5 %) presented active ulcers during treatment. Most published papers, however, do not include CEAP C6 patients,^11^^,^^14^^,^^15^ which constituted a notable aspect of this study. In Brazil, where approximately 75 % of the population (approximately 160 million individuals) depend exclusively on the care provided by the public health system, patient access to health services often involves long delays, especially for nonurgent conditions such as VVs. This may explain the greater proportion of individuals with more advanced stages of venous disease in the present study.^28^

Although varicose vein surgery poses a low risk for patients, sclerotherapy represents an even less invasive alternative, as it does not require hospitalization or invasive procedures.^29^ In this series, the authors found that patients selected for treatment for PFS were older than those who underwent CS, which aligns with findings from other available studies.^11^^,^^15^

In general, studies also indicate an immediate improvement in quality of life following interventions, whether through sclerotherapy or conventional surgery.^11^^,^^13^^,^^14^ However, extensive trials with long-term follow-up have shown a late deterioration in clinical severity and quality-of-life scores, typically occurring approximately two years after treatment,^12^^,^^15^ as CVD recurs. In this study, the authors observed an improvement in the quality-of-life scores of patients who underwent PFS and those who underwent CS. Nevertheless, the postoperative improvement was more substantial among patients who underwent surgery. These outcomes may be attributed to the inherent effects of the procedures themselves and the specific characteristics of the patients chosen for each treatment.

In the present study, pain was the factor exerting the most significant impact on the less favorable post-treatment outcomes observed in the foam group. These findings contrast with those of other studies, which suggest increased post-treatment pain following conventional surgery.^12^^,^^30^ The heightened perception of pain after sclerotherapy in the sample may be related to the effects of polidocanol, which can induce phlebitis due to local inflammatory reactions.^31^

In addition, the occurrence of phlebitis and hyperpigmentation potentially associated with foam application^13^ may correlate with the less favorable results observed in the post-procedure VEINES score quality-of-life domain, influencing patients' concern about the appearance of their legs.

Finally, patients’ individual characteristics inevitably influence their perception of postoperative improvement. Patients in the foam group tended to be older and to present more comorbidities. Previous studies have independently associated these two aspects with lower quality-of-life scores specific to varicose veins, irrespective of the severity of venous disease.^25^^,^^32^, ^33^, ^34^ Furthermore, in many circumstances, the age of patients and the severity of comorbidities (such as obesity) may be contraindications to traditional surgical procedures but not to sclerotherapy. Therefore, especially in the public health system, many patients (who already have a lower quality of life due to their clinical characteristics) end up receiving sclerotherapy as their only interventional treatment option.

It is important to highlight that, despite these distinctions, both treatments proved effective in addressing VVs. Surgery yields positive outcomes in terms of technical aspects at both early and late follow-up^12^^,^^30^ and in clinical aspects^35^ On the other hand, sclerotherapy, while potentially requiring reoperations and presenting local side effects, remains highly practical and cost-effective. Sclerotherapy also entails lower costs, does not require hospitalization or periprocedural anesthesia, and allows a quicker return to normal activities.^36^

In a healthcare context such as the Brazilian public health system, where many patients contend with multiple comorbidities or are elderly ‒ factors that may render surgical intervention challenging or prohibitive ‒ sclerotherapy emerges as a viable and effective alternative.

Several factors limit this study. While patients were enrolled consecutively, there was no randomization process for the treatment groups. Since the study evaluated patients treated within the public health system, it was restricted to assessing available treatment options. Unfortunately, other currently available methods, such as ablative techniques, could not be explored.

Despite its limitations, this is, to the best of our knowledge, one of the few, if any, studies to address the quality-of-life outcomes of polidocanol sclerotherapy versus surgical treatment using PROMs within one of the largest public health systems in the world.

## Conclusion

Both PFS and CS have demonstrated an immediate (within 30 days) positive impact on the quality of life of patients with VV.

Nevertheless, the improvement in quality-of-life scores attributed to surgical treatment surpasses that achieved through sclerotherapy, primarily because patients treated with foam tend to experience more pronounced pain and heightened aesthetic concerns.

## Authors’ contributions

**Lissa Severo Sakugawa:** Data curation, writing - original draft, writing – review & editing. **Felipe Soares Oliveira Portela:** Data curation, formal analysis, writing – original draft, writing – review & editing. **Andressa Cristina Sposato Louzada:** Data curation, methodology, validation. **Maria Fernanda Cassino Portugal:** Writing – review & editing, investigation. **Marcelo Passos Teivelis:** Writing – review & editing, project administration. **Cynthia de Almeida Mendes:** Investigation, formal analysis. **Lucas Lembrança Pinheiro:** Conceptualization, methodology, project administration. **Marcelo Fiorelli Alexandrino da Silva:** Methodology, resources, visualization. **Alexandre Fioranelli:** Conceptualization, project administration, supervision. **Nelson Wolosker:** Conceptualization, supervision, writing – review & editing.

## Conflicts of interest

The authors declare no conflicts of interest.

## Appendix Supplementary materials

Image, application 1
