# Supplementary material for: Polidocanol-foam treatment of varicose veins: Quality-of-life impact compared to conventional surgery
Source: Clinics (Sao Paulo). 2024 Apr 4;79:100346. doi: 10.1016/j.clinsp.2024.100346 (PMC11004700; doi:10.1016/j.clinsp.2024.100346)
Supplement: Supplementary file 1 [file mmc1.docx]

CLINICS-D-23-00530_Supplementary Material

**Appendix 1**

| **1.1. VCSS score[17]** | | | | |
| --- | --- | --- | --- | --- |
| **Attribute** | **Absent = 0** | **Mild = 1** | **Moderate = 2** | **Severe = 3** |
| Pain | None | Occasional, not restricting activity or requiring analgesics | Daily, moderate activity limitation, occasional analgesics | Daily, severe limiting activities or requiring regular use of analgesics |
| Varicose veins | None | Few, scattered: branch VVs | Multiple: GS varicose veins confined to calf or thigh | Extensive: Thigh and calf or GS and LS distribution |
| Venous edema | None | Evening ankle edema only | Afternoon edema, above ankle | Morning edema above ankle and requiring activity change, elevation |
| Skin pigmentation | None or focal, low intensity (tan) | Diffuse, but limited in area and old (brown) | Diffuse over most of gaiter distribution (lower 1/3) or recent pigmentation (purple) | Wider distribution (above lower 1/3) and recent pigmentation |
| Inflammation | None | Mild cellulitis, limited to marginal area around ulcer | Moderate cellulitis, involves most of gaiter area (lower 1/3) | Severe cellulitis (lower 1/3 and above) or significant venous eczema |
| Induration | None | Focal, circummalleolar (<5-cm) | Medial or lateral, less than lower third of leg | Entire lower third of leg or more |
| No. of active ulcers | 0 | 1 | 2 | >2 |
| Active ulceration, duration | None | <3 mo | >3 mo, <1y | Not healed >1y |
| Active ulcer, size | None | <2-cm diameter | 2- to 6-cm diameter | >6-cm diameter |
| Compressive therapy | Not used or not compliant | Intermittent use of stockings | Wears elastic stockings most days | Full compliance: stockings + elevation |

| **1.2 VEINES-QoL/Sy[18]** |
| --- |
| 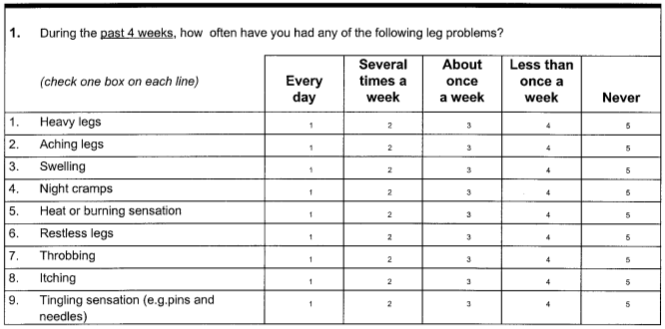 |
| 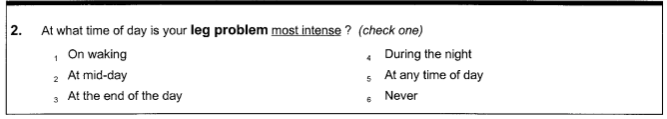 |
| 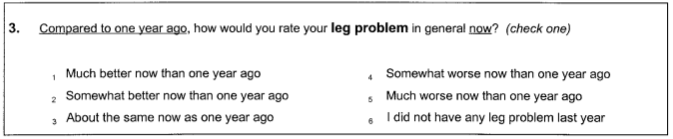 |
| 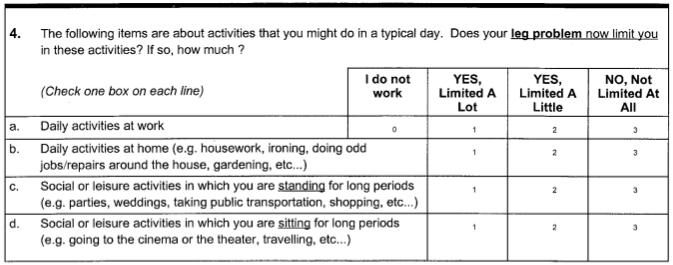 |
| 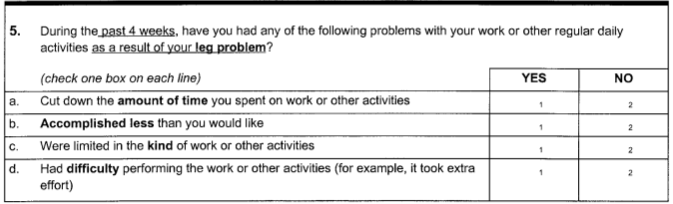 |
| 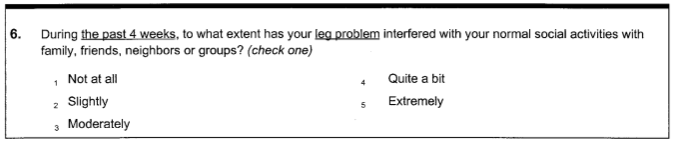 |
| 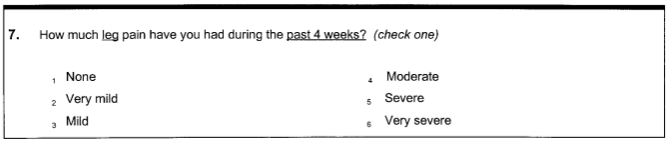 |
| 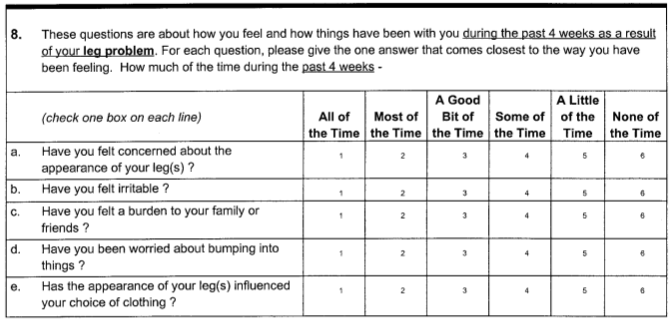 |

**Appendix 2** Multivariate analysis of factors that can influence quality-of-life scores Multiple linear regression.

| **2.1. VCSS** | | | | | | | |
| --- | --- | --- | --- | --- | --- | --- | --- |
| **Variable** | **Coefficient** | **Standard Error** | **95% CI** | | **Test statistics (Wald)** | **gl** | **p** |
|  |  |  | **Inferior** | **Superior** |  |  |  |
| Intercept | 5.34 | 1.86 | 1.70 | 8.97 | 8.27 | 1 | **0.004** |
| Sex (male) | 1.25 | 0.70 | -0.12 | 2.61 | 3.21 | 1 | 0.073 |
| Group (Foam) | 2.91 | 0.76 | 1.42 | 4.40 | 14.59 | 1 | **<0.001** |
| Hypertension | 0.52 | 0.69 | -0.83 | 1.86 | 0.57 | 1 | 0.451 |
| DM | -0.77 | 0.95 | -2.63 | 1.09 | 0.67 | 1 | 0.415 |
| Smoking | 1.70 | 1.32 | -0.89 | 4.29 | 1.66 | 1 | 0.198 |
| Physical activity | -0.45 | 0.72 | -1.85 | 0.96 | 0.39 | 1 | 0.532 |
| Obesity G3 | 4.23 | 2.41 | -0.50 | 8.96 | 3.08 | 1 | 0.079 |
| Obesity G2 | -0.30 | 1.33 | -2.92 | 2.31 | 0.05 | 1 | 0.820 |
| Obesity G1 | 0.45 | 0.88 | -1.27 | 2.17 | 0.26 | 1 | 0.607 |
| Overweight | 0.25 | 0.72 | -1.17 | 1.66 | 0.12 | 1 | 0.733 |
| Eutrophic | Ref. |  |  |  |  |  |  |
| Age (years) | 0.03 | 0.03 | -0.03 | 0.08 | 0.99 | 1 | 0.320 |
| VSCC pre | 0.42 | 0.06 | 0.30 | 0.54 | 46.53 | 1 | **<0.001** |
| **2.2. VEINES-QoL** | | | | | | | |
| **Variable** | **Coefficient** | **Standard Error** | **95% CI** | | **Test statistics (Wald)** | **gl** | **p** |
|  |  |  | **Inferior** | **Superior** |  |  |  |
| Intercept | 85.83 | 4.45 | 77.11 | 94.55 | 372.00 | 1 | **<0.001** |
| Sex (male) | 1.78 | 1.80 | -1.75 | 5.30 | 0.98 | 1 | 0.323 |
| Group (Foam) | -6.35 | 1.96 | -10.19 | -2.51 | 10.49 | 1 | **0.001** |
| Hypertension | -1.18 | 1.79 | -4.70 | 2.34 | 0.43 | 1 | 0.510 |
| DM | 5.78 | 2.51 | 0.86 | 10.70 | 5.30 | 1 | **0.021** |
| Smoking | 0.05 | 3.52 | -6.86 | 6.95 | 0.00 | 1 | 0.989 |
| Physical activity | 2.89 | 1.82 | -0.67 | 6.46 | 2.53 | 1 | 0.112 |
| Obesity G3 | -2.43 | 5.24 | -12.70 | 7.83 | 0.22 | 1 | 0.643 |
| Obesity G2 | -18.02 | 3.05 | -23.99 | -12.04 | 34.93 | 1 | **<0.001** |
| Obesity G1 | -2.74 | 2.27 | -7.19 | 1.71 | 1.46 | 1 | 0.228 |
| Overweight | -2.45 | 1.81 | -6.00 | 1.11 | 1.82 | 1 | 0.177 |
| Eutrophic | Ref. |  |  |  |  |  |  |
| Age (years) | -0.08 | 0.07 | -0.22 | 0.05 | 1.48 | 1 | 0.223 |
| **2.3. VEINES-Sym** | | | | | | | |
| **Variable** | **Coefficient** | **Standard Error** | **95% CI** | | **Test statistics (Wald)** | **gl** | **p** |
|  |  |  | **Inferior** | **Superior** |  |  |  |
| Intercept | 87.02 | 5.86 | 75.54 | 98.49 | 220.81 | 1 | **<0.001** |
| Sex (male) | 2.15 | 2.37 | -2.49 | 6.79 | 0.83 | 1 | 0.363 |
| Group (Foam) | -10.71 | 2.58 | -15.76 | -5.65 | 17.23 | 1 | **<0.001** |
| Hypertension | -1.00 | 2.36 | -5.62 | 3.63 | 0.18 | 1 | 0.673 |
| DM | 3.98 | 3.30 | -2.49 | 10.46 | 1.45 | 1 | 0.228 |
| Smoking | -0.93 | 4.63 | -10.01 | 8.15 | 0.04 | 1 | 0.841 |
| Physical activity | 3.57 | 2.39 | -1.13 | 8.26 | 2.22 | 1 | 0.136 |
| Obesity G3 | -6.81 | 6.89 | -20.32 | 6.70 | 0.98 | 1 | 0.323 |
| Obesity G2 | -16.69 | 4.01 | -24.56 | -8.83 | 17.32 | 1 | **<0.001** |
| Obesity G1 | -4.10 | 2.99 | -9.95 | 1.75 | 1.89 | 1 | 0.170 |
| Overweight | -2.91 | 2.39 | -7.59 | 1.76 | 1.49 | 1 | 0.222 |
| Eutrophic | Ref. |  |  |  |  |  |  |
| Age (years) | -0.05 | 0.09 | -0.22 | 0,13 | 0.26 | 1 | 0.610 |
